# Perfectionism and Obsessive-Compulsive Symptom Dimensions in University Students

**DOI:** 10.7759/cureus.109940

**Published:** 2026-05-30

**Authors:** Yana Quarantini-Alvim, Monicke O Lima, Raíza Alves-Pereira, Renan B de Christo, Gustavo S Alves, Luna B Dib, David G Roiter, Fernanda Cupertino-Silva, Lucas C Quarantini, Aline S Sampaio

**Affiliations:** 1 Psychiatry, Hospital Universitário Professor Edgard Santos, Universidade Federal da Bahia, Salvador, BRA; 2 Psychiatry, Hospital das Clínicas da Faculdade de Medicina da Universidade de São Paulo (HCFMUSP), São Paulo, BRA; 3 Medicine, Hospital Juliano Moreira, Universidade Federal da Bahia, Salvador, BRA; 4 Medicine, Universidade Federal da Bahia, Salvador, BRA; 5 Psychology, Faculdade Santa Casa de Misericordia da Bahia, Salvador, BRA; 6 Medicine, Hospital Universitário Professor Edgard Santos, Universidade Federal da Bahia, Salvador, BRA

**Keywords:** compulsive, obsessive-compulsive symptoms, obsessive thoughts, perfectionism, university students

## Abstract

Introduction

Perfectionism is a multidimensional and transdiagnostic construct associated with several psychiatric conditions, including obsessive-compulsive disorder (OCD). The relationship between perfectionism and specific obsessive-compulsive symptom (OCS) dimensions remains unclear. This study aims to investigate the correlation between perfectionism and obsessive-compulsive symptom dimensions in a non-clinical sample of university students from different academic fields.

Methods

A cross-sectional study was conducted with undergraduate students from the Federal University of Bahia (UFBA), Brazil. Participants completed self-report instruments assessing the Multidimensional Perfectionism Scale - Short Form (MPS-SF), the Dimensional Yale-Brown Obsessive-Compulsive Scale - Short Version (DYBOCS-SV), the Generalized Anxiety Disorder-7 (GAD-7), and the Patient Health Questionnaire-9 (PHQ-9). Correlational analyses were performed using the Spearman test, and multiple linear regression was conducted to evaluate the OCS dimensions, anxiety, and depression on the variance in perfectionism scores.

Results

The sample demonstrated moderate to high levels of perfectionism, and approximately 28% of participants reported clinically relevant OCS. Perfectionism was significantly associated with anxiety symptoms and with some specific OCS dimensions. Depressive symptoms were not significantly associated with perfectionism in the regression model.

Conclusion

These findings suggest that perfectionism is associated with specific OCS dimensions and anxiety symptoms in a non-clinical university population. The results support the relevance of investigating perfectionism within a dimensional and transdiagnostic framework, particularly in relation to subclinical obsessive-compulsive phenomena during emerging adulthood.

## Introduction

Perfectionism is broadly defined as a personality trait characterized by a constant pursuit of perfect and high standards, involving self-criticism and criticism of others. Also, perfectionism is multidimensional and can be divided into three forms: self-oriented (excessive concern with being perfect and highly critical when failing to meet one's demands), other-oriented (the belief that others should be perfect), and socially prescribed (excessive concern with being socially perfect, with the belief that failing to meet external demands will result in severe criticism) [1,2]. Considering its excessive presentation, perfectionism can affect various areas of life, such as academic performance, interpersonal relationships, and mental health [3].

In this context, perfectionism has been widely conceptualized not only as a relatively stable personality trait but also as a transdiagnostic phenomenon. Studies have identified perfectionism as a risk factor, protective factor, or a factor in the perpetuation of existing symptoms [4-6]. Several studies have investigated the relationship between perfectionism and psychiatric conditions, such as anxiety disorders [7,8], depression disorders [9], and obsessive-compulsive disorder (OCD) [5]. OCD is characterized by the presence of obsessions (intrusive, recurrent thoughts, images, or urges) and/or compulsions (repetitive behaviors or mental acts performed to reduce distress or prevent a feared event) [10], which can significantly impair functioning and quality of life [11]. However, the relationship between perfectionism and the specific dimensions of obsessive-compulsive symptoms remains unclear.

Although OCD has been investigated as a categorical diagnosis, there is also growing evidence that suggests its symptoms are organized along distinct symptom dimensions (checking/aggression, ordering/symmetry, contamination/cleaning, religious, sexual, and hoarding), each potentially reflecting partially different cognitive and neurobiological mechanisms [12]. These dimensions appear to be associated with differential patterns of neural activation and distinct personality profiles, supporting the hypothesis that different neurobiological substrates underlie each symptomatic domain [13]. Consistent with this, a previous study has observed that perfectionism is more consistently associated with specific dimensions of obsessive-compulsive symptoms (OCS), particularly symmetry, ordering, and checking symptoms [14], rather than with obsessive-compulsive psychopathology as a whole. These findings lead us to hypothesize that certain dimensions of OCS may be more correlated with perfectionism than others.

Based on this dimensional perspective, also regarding severity, the value of studying obsessive-compulsive symptoms (OCS) instead of OCD diagnosis is supported by familial studies, which have documented robust genetic overlap and shared biological and behavioral characteristics between individuals diagnosed with OCD and their undiagnosed relatives, suggesting a familial predisposition along a continuum of severity [15-17]. Sreeraj et al. (2021), in a study involving 1406 individuals from 481 Indian multiplex families, identified a significant difference in the prevalence of OCS among first-degree relatives of OCD probands, compared with first-degree relatives of probands with other mental disorders of control probands, indicating that psychopathological vulnerability may manifest in a subclinical manner even in the absence of a formal diagnosis [18]. In line with these findings, Lima et al. (2024), in a sample of 994 Brazilian OCD families, compared families in which only the proband had OCD (simplex) with families in which at least one more family member had OCD or OCS (multiplex) and found that probands from multiplex families had earlier age at OCD onset, more illness duration, more sexual, religious and hoarding OC symptoms, as well as a higher frequency of comorbidities with OCD-related disorders [19]. From this perspective, these findings highlight the relevance of investigating OCS in non-clinical populations. 

The entrance to university usually coincides with a transition into adulthood. This is a particularly vulnerable phase of human development, characterized by a higher incidence of various mental health problems [20] such as post-traumatic stress disorder [21-24], suicide attempts [25], depression [26], experiential avoidance [27], and social anxiety [7]. During this transitional phase, there is also a second peak in OCD incidence, with a slight female predominance [28]. Previous studies with university students reported that perfectionism has also been associated negatively with psychological well-being [29] and academic performance [30], highlighting the importance of investigating this group.

This study aims to investigate the correlation between perfectionism and obsessive-compulsive symptom dimensions in a non-clinical sample of university students from different academic fields.

## Materials and methods

The sample for this study consisted of university students from the Federal University of Bahia (UFBA), Brazil. The study was approved by the Institutional Review Board of the UFBA Medical School (protocol number: 47131221.0.0000.5577) and adhered to the ethical principles of the Declaration of Helsinki (2013) and the Brazilian National Health Council Resolution 466/12. Data collection occurred from September 2020 to March 2021, when students were taking online classes due to the COVID-19 pandemic. Following approval, an invitation to participate in the study was sent to all directories and academic committees of each academic field, requesting that they forward our link to each student's email. After giving consent, students completed the self-administered instruments online via the Google Forms platform (Google, Mountain View, California).

The Multidimensional Perfectionism Scale-Short form (MPS-SF) is a 15-item measure that assesses perfectionism across three dimensions: self-oriented, other-oriented, and socially prescribed, with five items assessing each dimension. Participants responded to the items using a Likert scale ranging from 1 = strongly disagree to 7 = strongly agree, where higher scores indicate higher levels of perfectionism [31]. The Dimensional Yale-Brown Obsessive-Compulsive Scale Short Version (DY-BOCS-SV) is a derived instrument from the Dimensional Yale-Brown Obsessive-Compulsive Scale, adapted by the DY-BOCS author herself, Rosario-Campos, that measures the presence and severity of OCS in six different dimensions: checking/aggression, ordering/symmetry, contamination/cleaning, religious, sexual, and hoarding. The DY-BOCS-SV comprises 15 items, and symptom severity is rated on a Likert scale ranging from 0 = minimal to 5 = very severe [32]. To evaluate anxiety, the Generalized Anxiety Disorder-7 (GAD-7) was used as a self-reported scale to assess the severity of generalized anxiety disorder symptoms through seven items that evaluate common symptoms, such as being nervous or anxious, unable to control concern, worrying excessively about various things, having problems relaxing, being uneasy, easily irritable, or having excessive fear over the past two weeks. Each GAD-7 item is scored on a four-point scale, ranging from 0 (not at all) to three (nearly every day), with total scores from 0 to 21 indicating more severe symptoms and demonstrating high sensitivity and specificity for the diagnosis of general and other anxiety disorders. The interpretation of the results is based on the sum of the scores assigned to the seven items, ranging from 0 to 21. The severity ranges are: 0-4 (no significant anxiety), 5-9 (mild anxiety), 10-14 (moderate anxiety), and 15-21 (severe anxiety). Scores ≥10 indicate the presence of clinically relevant anxiety symptoms [33]. Finally, the Patient Health Questionnaire-9 (PHQ-9) was utilized as a self-report measure for assessing depression severity through nine items focusing on symptoms such as low mood, loss of interest, changes in sleep and appetite, and thoughts of death or self-harm. Respondents rate each PHQ-9 item based on frequency over the last two weeks using a scale from 0 (not at all) to three (nearly every day), resulting in a total score from 0 to 27 where higher scores indicate higher severity of depression symptoms, as mild (5-9), moderate (10-14), moderately severe (15-19), and severe (20-27); scores ≥10 indicate the presence of clinically relevant depressive symptoms [34]. All instruments used in this study are either in the public domain or were used for academic research purposes in accordance with the authors' original publication guidelines. The DY-BOCS-SV is a shortened version adapted by the author of the original scale herself (Rosario-Campos).

For the statistical analysis performed in the sample, the scores for each dimension of OCS and the scores for perfectionism were evaluated. Considering perfectionism as a multidimensional continuous construct, no categorical cutoff points were adopted for the MPS-SF. Therefore, score interpretation was based on percentile analysis of the sample distribution. The Shapiro-Wilk test was used to test the data distribution. Since the data did not follow a normal distribution, the Spearman's correlation test was used with perfectionism and dimensions of OCS (correlation coefficient from 0.00 to 0.10 indicates a correlation, 0.10-0.39 indicates a weak correlation, 0.40-0.69 indicates a moderate correlation, 0.70-0.89 indicates a strong correlation, and 0.90-1.00 indicates a very strong correlation) [35]. Multiple linear regression was used to investigate the influence of OCS dimensions (checking/aggression, ordering/symmetry, contamination/cleaning, religious, sexual, and hoarding), anxiety, and depressive symptoms in the variance of perfectionism. Perfectionism was defined as the dependent variable because it constitutes the central object of this study, with no intention of establishing temporal or causal precedence relationships among the variables. All independent variables were entered simultaneously into the model (Enter method). Given the high correlations observed in the Spearman correlation analysis, multicollinearity was assessed prior to the regression using the variance inflation factor (VIF) of < 5 and tolerance of > 0.2. Additional regression assumptions were also verified: Durbin-Watson values between 1.5 and 2.5 for error independence, and random dispersion in the standardized residual plot for homoscedasticity. All assumptions were satisfied. Statistical significance was defined as p < 0.05, and all analyses were conducted using software SPSS version 25.0 (IBM Inc., Armonk, New York).

## Results

A total of 389 students from a Brazilian public university responded to the questionnaires. The sample exhibited moderate to high levels of perfectionism in MPS-SF [31], with a median score of 68 (SD = 14). Percentile analysis revealed that the 25th percentile scored at 60, the 50th percentile at 68 (median), and the 75th percentile at 77. The median of perfectionism in each academic field was the following: physics, mathematics, and technology = 67.5; biological sciences and health professions = 65; philosophy and human sciences = 69; languages = 67; and arts = 71. 

The sample has shown mild to moderate anxiety scores and moderate depressive scores. Additionally, clinically relevant OCD symptoms (at least one OCS at severe or very severe intensity) were reported by approximately 28% of students. The median for obsessions was eight (SD = 5.80), compulsions was five (SD = 4.95), and the DY-BOCS-SV (total score) [32] score was median = 13 (SD = 10.17). The median and mean scores of the OCS dimensions indicated a higher score in aggression/checking and ordering/symmetry. The descriptive analysis is presented in Table 1.

**Table 1:** Sociodemographic characteristics and symptoms scores in students in Brazil (n = 389) MPS-SF - Multidimensional Perfectionism Scale-Short form [31]; DYBOCS-SV - Dimensional Yale-Brown Obsessive-Compulsive  Scale Short Form [32]; GAD-7 - Generalized Anxiety Disorder 7-item scale [33]; PHQ-9 - Patient Health Questionnaire-9 [34] Missing values were observed for age* (n = 1), gender identity** (n = 2), and ethnicity*** (n = 7)

Variable	Mean	N (%) / median (SD)
Participants		389 (100%)
Age, years*	25.89	23.00 (± 8.30)
Academic fields		
Physics, mathematics, and technology		106 (27.25%)
Biological sciences and health professions		75 (19.28%)
Philosophy and human sciences		130 (33.42%)
Languages		44 (11.31%)
Arts		34 (8.74%)
Occupation		
Student only		225 (57.84%)
Studying and working		163 (41.90%)
Medical leave/benefit		1 (0.25%)
Gender identity**		
Cisgender man		126 (32.55%)
Cisgender woman		248 (64.08%)
Transgender man		2 (0.51%)
Transgender woman		1 (0.25%)
Others		10 (2.58%)
Ethnicity***		
Black		236 (60.7%)
White		139 (35.7%)
Indigenous		3 (0.8%)
Asian		2 (0.5%)
Others		2 (0.5%)
MPS-SF	67.94	68.00 (±14.00)
GAD-7	9.37	9.00 (±5.52)
PHQ-9	12.63	12.00 (±6.70)
DY-BOCS-SV	13.90	13.00 (±10.17)
Aggression/checking	4.47	5.00 (± 3.22)
Religious	1.52	0.00 (±2.53)
Sexual	1.84	0.00 (±2.64)
Ordering/symmetry	2.59	2.00 (±2.87)
Contamination/cleaning	2.08	0.00 (±2.76)
Hoarding	1.39	0.00 (±2.20)
Total obsessions	8.29	8.00 (±5.80)
Total compulsions	5.61	5.00 (±4.95)

The Spearman's correlation analysis indicated a strong and significant relationship between perfectionism and the checking/aggression dimension (p < 0.001). Other OCD symptom dimensions showed weak but statistically significant correlations: ordering/symmetry (p < 0.001), contamination/cleaning (p <0.001), sexual (p = 0.035), religious (p = 0.002), and hoarding (p = 0.007). These results are summarized in Table 2. 

**Table 2:** Spearman correlation between perfectionism and obsessive-compulsive symptom dimensions Obsessive-compulsive symptom dimensions scores (checking/aggression, ordering/symmetry, contamination/cleaning, religious, sexual, and hoarding) were assessed using the Dimensional Yale-Brown Obsessive-Compulsive Scale Short Form (DYBOCS-SV) [32]; Statistical significance was defined as p <0.05.

Dimensions	Spearman's rho	p-values
Aggression/checking	0.730	<0.001
Religious	0.156	0.002
Sexual	0.107	0.035
Ordering/symmetry	0.392	<0.001
Contamination/cleaning	0.218	<0.001
Hoarding	0.136	0.007
Total obsessions	0.359	<0.001
Total compulsions	0.389	<0.001
DYBOCS-SV (total score)	0.359	<0.001

Multiple linear regression revealed that the model was statistically significant (F(8, 379) = 18.173, p < 0.001), explaining 27.7% of the variance in perfectionism (R² = 0.277, adjusted R² = 0.262). All multicollinearity assumptions were met, with VIF values ranging from 1.171 to 2.420 and Tolerance values above 0.2. The Durbin-Watson statistic (2.046) indicated independence of errors. Among the variables entered in the model, anxiety symptoms (GAD-7; β = 0.301, p < 0.001), ordering/symmetry (β = 0.253, p < 0.001), and checking/aggression (β = 0.119, p = 0.032) significantly influenced perfectionism variance. Depression symptoms (PHQ), religious, sexual, contamination/cleaning, and hoarding dimensions did not remain associated with perfectionism. These results are presented in Table 3. 

**Table 3:** Multiple linear regression analysis: associations between OCS dimensions, anxiety, and depressive symptoms with perfectionism R² = 0.277; Adjusted R² = 0.262; F(8, 379) = 18.173, p < 0.001. SE - standard error; β - standardized coefficient; CI - confidence interval; VIF - variance inflation factor; OCS - obsessive-compulsive symptoms; MPS-SF - Multidimensional Perfectionism Scale-Short form [31]; DYBOCS-SV - Dimensional Yale-Brown Obsessive-Compulsive Scale Short Form [32]; GAD-7 - Generalized Anxiety Disorder 7-item scale [33]; PHQ-9 - Patient Health Questionnaire-9 [34] OCS dimensions scores (checking/aggression, ordering/symmetry, contamination/cleaning, religious, sexual, and hoarding) were assessed using the Dimensional Yale-Brown Obsessive-Compulsive Scale Short Form (DYBOCS-SV). Variable dependent was the Multidimensional Perfectionism Scale-Short form score (MPS-SF).

Variable	B	SE	β	t	p	95% CI	VIF
(Constant)	55,582	1347		41,250	0.000	52.933-58.231	
GAD-7	0.741	0.167	0.301	4436	0.000	0.413-1.070	2.420
PHQ-9	0.050	0.135	0.025	0.373	0.709	-0.215-0.316	2.331
Checking/aggression	0.500	0.233	0.119	2150	0.032	0.043-0.957	1.596
Religious	-0.079	0.271	-0.015	-0.292	0.770	-0.612- 0.454	1.337
Sexual	-0.396	0.262	-0.077	-1509	0.132	-0.911- 0.120	1.360
Ordering/symmetry	1199	0.233	0.253	5136	0.000	0.740-1.658	1.273
Contamination/cleaning	0.252	0.237	0.051	1062	0.289	-0.214-0.717	1.214
Hoarding	-0.053	0.292	-0.009	-0.183	0.855	-0.627-0.520	1.171

## Discussion

This study explored the relationship between perfectionism and dimensions of obsessive-compulsive symptoms (OCS). In our sample, composed of students from various academic fields, the mean perfectionism score was 67.94 (SD = 14.00), indicating a moderate to high level. This value stands out in comparison to other studies that have used the MPS-SF [31]. Wang et al. (2022) analyzed a sample of university students and reported a mean score of 45.26 (SD = 7.66) [7], while another study conducted by Wang and Wu (2021) with medical students found a mean of 40.54 (SD = 10.94) [36]. Similarly, an Italian study by Iaia et al. (2024) involving students with specific learning disorders reported a mean score of 20.26 (SD = 1.13) [37]. These discrepancies suggest that Brazilian students exhibit higher levels of perfectionism compared to these samples of university populations. One possible explanation is that the sample was recruited from a public university with a highly competitive admission process, which may attract students with greater self-demand and higher academic performance expectations.

Regarding OCS prevalence, in our sample, approximately 28% of students reported experiencing at least one symptom at a severe or very severe intensity of OCS. The reported prevalence of OCD within the general population is about 1-3% [38], and the prevalence of OCS is much higher (21-25%) [39]. Our study found a higher frequency compared with the general population. Considering that this sample involved only University students, the findings were not so different from those of other authors. A Chinese study involving 810 medical students found that 50.5% of the sample exhibited OCS [40], which may be attributed to high familial, social, and self-expectations. Similarly, another study in China, also conducted during the COVID-19 pandemic, detected a high percentage of OCS among university students [41]. In Spain, two studies identified the findings: one showed that female university students scored significantly higher on OCS compared to the average for women of all ages [42], and the other found that 30% of the university student sample had high scores for OCS [43]. These studies suggest that OCS may be prevalent among the population entering university and seems to be linked to mental health problems and impaired social functioning. Although slightly higher than expected when compared with the frequency in the general population, our finding is consistent with the literature in the population of students. 

The high frequency of OCS may also be related to the instrument used. This trend has already been reported by another study that employed the DY-BOCS-SV in mothers of elementary school children, who obtained a mean global score of 3.6 (SD = 6.9), with 40% of the sample presenting at least one OCS symptom [44]. It is important to consider that the instrument used, a shortened version of the DY-BOCS, may have influenced these high scores, especially given that it has not been specifically validated for university student populations.

All variables evaluated in Spearman's correlation were related to perfectionism. The inclusion of different DY-BOCS groupings, such as total score, obsessions, compulsions, and symptom dimensions, may have introduced redundancy in the results. The regression analysis examined the effect of obsessive-compulsive symptom dimensions, anxiety, and depression on perfectionism variance, and the results indicated that the dimensions of checking/aggression and ordering/symmetry were significantly related to perfectionism. Among these, the checking/aggression dimension exhibited the highest median severity score, followed by ordering/symmetry. These findings are consistent with previous studies, identifying an association between perfectionism and the checking and ordering dimensions of OCS [21,45]. In other studies using non-clinical college samples, perfectionism was associated with the checking symptom dimension of OCS, and the relation with the ordering/symmetry dimension was not found [46,47]. However, this prior study was conducted specifically in psychology student samples, while our study focused on a more diverse sample of university students from various academic fields. 

Similarly with previous studies [8,48], anxiety symptom scores were significantly related with perfectionism. Although the present study did not directly assess intolerance of uncertainty (IU), previous research suggested that IU is a mediator between OCS, perfectionism, and anxiety [49,50]. Notably, IU acts as a mediating variable between perfectionism and obsessive-compulsive symptoms [51], and it is associated with specific behaviors such as checking, ordering, and symmetry, in addition to obsessive symptoms in non-clinical university samples [52], reinforcing its relevance in understanding OCS in these samples. Thus, it is possible that the findings of the present study were influenced by IU as an unmeasured mediating variable, underscoring the need for future investigations to further explore the underlying mechanisms linking perfectionism and the various dimensions of OCS. 

Among the limitations, the recruitment was conducted indirectly via email. This method did not allow us to determine the proportion of people who were invited but chose not to respond to the survey. Also, through the invitation email, participants had access to the project title 'Assessment of Obsessive-Compulsive Symptoms in University Students'. The propensity to participate in the study could be higher for students who were already familiar with the term obsessive-compulsive symptoms. Another limitation was the online application of the questionnaire. However, a previous study [53] stated that regardless of the administration method, the results were equivalent. 

Another limitation is that the data collection period, during the COVID-19 pandemic, may have influenced the anxiety levels observed in the sample. In our study, participants presented moderate anxiety levels according to the GAD-7, which is consistent with the study by Silva et al. 2023, also conducted during the COVID-19 period in a Brazilian non-clinical sample, which identified a predominance of moderate and severe anxiety levels in the general population [54]. Finally, the instrument used to quantitatively assess different dimensions of OCS, the DYBOCS-SV, is a short version of DYBOCS, and it has been used in a non-clinical population [43] by the instrument's author. Acknowledging the limitation of the instrument to attribute diagnosis, it was used in the study only for evaluating the dimensions as continuous variables, and the analysis focused on correlating each dimension score with perfectionism. Moreover, the study focused on analyzing these correlations, without trying to establish any causal relationship.

## Conclusions

Our findings should be interpreted as exploratory, and further studies are required. The significant associations observed between perfectionism and OCS checking/aggression and ordering/symmetry dimensions suggest that perfectionism may be present even in subclinical expressions of obsessive-compulsive phenomena, beyond the severity required for a formal diagnosis of OCD, and that it could be more related to some specific OCS dimensions. The contribution of these findings to the phenomenological characterization of OCD subgroups may improve clinical understanding and help guide future research involving more homogeneous subgroups.
